# TRACKING CHANGES IN MULTIMORBIDITY AMONG RACIALLY AND ETHNICALLY DIVERSE POPULATIONS

**DOI:** 10.1093/geroni/igz038.1285

**Published:** 2019-11-08

**Authors:** Ana R Quiñones, Anda Botoseneanu, Sheila Markwardt, Corey Nagel, Jason T Newsom, David A Dorr, Heather G Allore

**Affiliations:** 1 Family Medicine, Oregon Health & Science University, Portland, Oregon, United States; 2 University of Michigan, Dearborn, Michigan, United States; 3 Oregon Health & Science University, Portland, Oregon, United States; 4 University of Arkansas for Medical Sciences, Little Rock, Arkansas, United States; 5 Portland State University, Portland, Oregon, United States; 6 Yale University, New Haven, Connecticut, United States

## Abstract

Multimorbidity is widely recognized as having adverse effects on health and wellbeing above and beyond the risk attributable to individual chronic disease. Much of what is known about multimorbidity rests on research that has largely focused on one point-in-time, or from a static perspective, with little consideration to issues involved in assessing longitudinal changes in multimorbidity. In addition, less focus has been placed on assessing racial and ethnic variations in longitudinal changes of multimorbidity. Addressing this knowledge gap, we highlight important issues and considerations in addressing multimorbidity research from a longitudinal perspective and present findings from longitudinal models that examine differences in the rate of chronic disease accumulation and multimorbidity onset between non-Hispanic white (white), non-Hispanic black (black), and Hispanic study participants in the Health and Retirement Study starting in middle-age and followed for up to 16 years.

